# Counterpropagating interactions of self-focusing Airy beams

**DOI:** 10.1038/s41598-019-41418-4

**Published:** 2019-03-21

**Authors:** Nicolas Marsal, Noémi Wiersma, Marc Sciamanna, Delphine Wolfersberger

**Affiliations:** 1 Chaire Photonique, LMOPS, CentraleSupélec, Université Paris Saclay, 2 rue Edouard Belin, 57070 Metz, France; 2 Université de Lorraine, CentraleSupélec, Chaire Photonique, LMOPS, 2 rue Edouard Belin, 57070 Metz, France

## Abstract

We study the first experimental collisions of two incoherent self-focused counterpropagating Airy beams in a nonlinear crystal. Their interactions demonstrate that the self-focusing dynamics of the Airy beams can be spatially controlled by the counterpropagating Airy beam. By tuning the misalignment and the size of the beams, we can control the output position of the self-focused Airy beam and switch to multiple outputs. Also the nonlinearity strength enables to engineer the temporal stability of the resulting waveguide structure.

## Introduction

Since the first observation of an optical Airy beam in 2007, its peculiar propagation properties have raised much interest. The Airy beam propagates diffraction-free and along a parabolic accelerating trajectory with self-healing ability under linear conditions^1–3^. Contrary to the ideal Airy solution in quantum mechanics^4^, the optical Airy beam contains a finite energy and presents the shape-preserving accelerating propagation over a finite distance only. The linear Airy properties can be tuned via the main lobe beam waist and via the so-called truncation parameter^5^, but its shape and trajectory can also be controlled using a nonlinear propagation. While a weak focusing or a defocusing nonlinearity enable the Airy beam to self-trap and to preserve the Airy properties over a larger distance^6^, it has been demonstrated that a strong focusing nonlinearity leads to a break-down of the Airy beam into what has been called an off-shooting soliton (OSS) and a remaining accelerating structure^7–9^. Recently we have experimentally demonstrated the self-focusing properties of a 1D-Airy beam in a nonlinear photorefractive crystal^10^ and confirm the true solitonic property of the resulting OSS^11^.

The peculiar properties of the Airy beams have also motivated the study of multiple Airy beams interactions. In the co-propagating case, theoretical results suggest particle-like interactions under strong self-focusing conditions, where the phase and the beams’ distance enable the tuning of attractive or repulsive solitonic interactions^12–14^. Also the cross-interaction of weak focusing Airy beams allows for the formation of a straight-propagating solitonic beam^15^. Wider interaction possibilities are known to occur when considering not a co-propagating but a counter-propagating scheme^16^. In particular the mutual attractive interactions of incoherent counterpropagating beams enable to photoinduce joint waveguides, however only for misalignments smaller than two beam diameters^17^. So far in the counterpropagating (CP) configuration, theory predicts that two colliding Airy beams photoinduce multiple waveguides for all-optical routing even for strong misalignment, hence significantly contrasting with what is known for interacting Gaussian beams^18^.

In this paper, we present the first experimental observation of such interactions between two CP incoherent Airy beams when applying non linear focusing conditions. The study of the spatiotemporal dynamics of self-focused Airy beams in a photorefractive crystal shows that the presence of a CP Airy beam (backward beam) induces a more complex refractive index structure which modifies the trajectory of the forward Airy beam. By tuning the transverse shift and the beams’ size, the solitonic output beam can be shifted along a distance of several waists and/or split into multiple output beams. Furthermore, the life duration time of the photoinduced waveguide structure can be controlled via the photorefractive nonlinearity (through the externally applied voltage). These results demonstrate the possibility for photoinducing more complex and various waveguiding structures when using counterpropagating Airy beams instead of Gaussian beams. This result is achievable thanks to the Airy beams’ multi-scale dimensions and through their possible interactions in even strongly misaligned configurations^18,19^.

**Figure 1:**
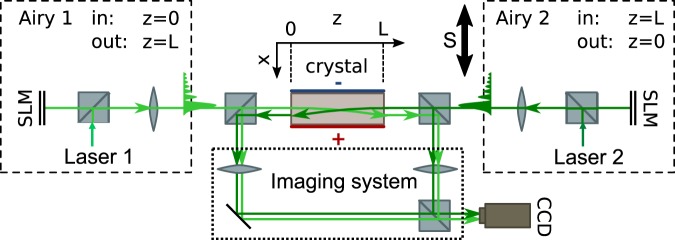
Study of two incoherent CP Airy beams in a biased fosing photorefractive (PR) crystal: experimental setup.

Figure 1 presents the experimental set-up used for studying the CP Airy beams interactions. Two incoherent CP Airy beams are injected in a biased photorefractive SBN-crystal (5*mm* * 5 *mm* * 1 cm). Both Airy beams propagate in opposite longitudinal *z*-directions and accelerate towards the +*x*-direction with the same parameters. The two different lasers have the same wavelength *λ* = 532 nm and are used with an optical power *P*_*A*_ = 60 *μ*W. We vary the Airy lobe’s size *x*_*A*_ (with a truncation parameter *a* ≈ 0.04) and the external electric voltage *U*_*SBN*_. The second Airy beam can be shifted along *x* direction to tune the alignment of the CP beams (via the control parameter called *S*). To measure the spatial evolution of the Airy beams at both sides of the crystal, we use two imaging systems with lenses and a CCD-camera.

In this paper we will analyze the beam intensity profiles at the output side *z* = *L* of the crystal to characterize the mutual interactions between the Airy beams. Due to symmetry of our system, the output profiles observed at *z* = 0 will be similar. As shown on Fig. 1, two intensity profiles (at *z* = *L*) are superimposed on the camera: the forward Airy beam (named “Airy 1”) output after propagating through the medium and the reflection of the backward Airy beam (named “Airy 2”) before entering in the sample. The observation of Airy beam 2 reflection enables us to compare the output position of the forward beam with respect to Airy beam 2 lobes: it will allow to identify the attraction force of the Airy beam 2 on the forward beam 1 through the multi-lobes’ photoinduced structure inside the crystal.

## Method and Results

### Comparison between one single Airy beam propagation and two Airy beams counterpropagation

**Figure 2:**
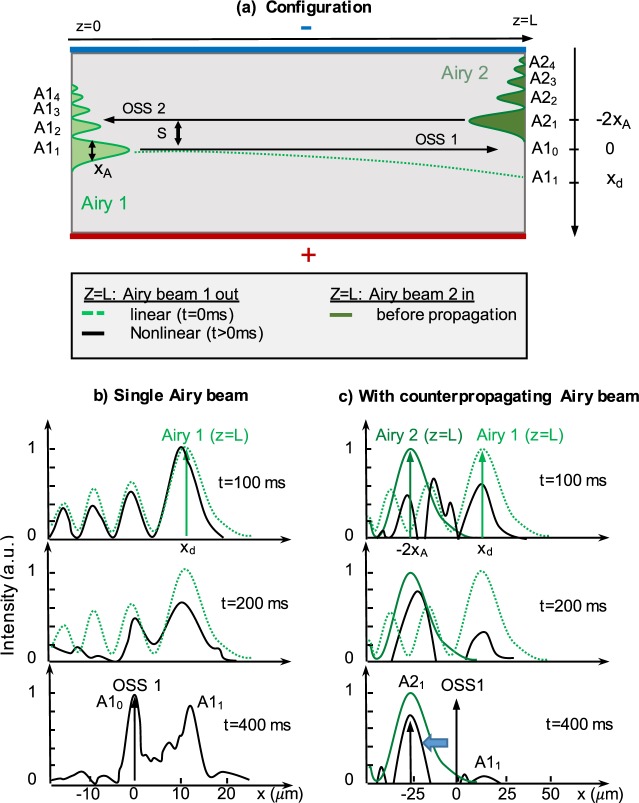
Influence of a CP Airy beam on the Off-Shooting Soliton spatial position. (**a**) Experimental configuration scheme. (**b**,**c**) Observation of the transverse output profiles at *z* = *L* [Fig. 1] versus time (**b**) when one single Airy beam propagates in the PR crystal (**c**) when an additional counterpropagating Airy beam is injected. Solid green lines correspond to what we called “Airy beam 2 in”, dashed green lines to the “linear Airy beam 1 out” (after propagation) and the solid black lines to the “nonlinear Airy beam 1 out” (after propagation). The numbering *A*1_1_, *A*1_2_... and *A*2_1_, *A*2_2_... correspond to the different lobes of Airy beam 1 and 2 and consequently to the different inputs/outputs for optical interconnects.

The first experiment using CP Airy beams is realized with parameters *x*_*A*_ = 14 *μ*m and *U*_*SBN*_(*t* > 0 *s*) = 2 kV. Figure 2 shows, in the experimental configuration illustrated in [Fig. 2(a)], a comparison of the spatial output profiles of the self-focused forward Airy beam 1 propagating along the +*z*-direction without [Fig. 2(b)] and with a CP Airy beam 2 (*S* = −2*x*_*A*_ = −28 *μ*m) [Fig. 2(c)]. We analyse the spatial output position of the self-focused Airy beam 1. As detailed in ref.^10^, a single propagating Airy beam under strong nonlinear focusing condition turns into a solitonic beam (off-shooting soliton or OSS) at the transverse zero-deflection position [*A*1_0_ in Fig. 2(a)], that coexists with an accelerating beam structure [*A*1_1_ at *z* = *L* in Fig. 2(a)]. This dynamics is experimentally well reproduced in Fig. 2(b), where the intensity is first attracted towards *x*_*d*_ = 12 *μ*m (linear deflection position at *t* = 100 ms). At *t* = 400 ms we observe a high and narrow intensity peak at the reference zero deflection position *x* = 0 *μ*m (OSS1) and the accelerating beam starts forming again its multi-lobe structure around the initial linear deflection position *x*_*d*_ (coexistence between the OSS and the accelerating structure).

The injection of CP Airy beam 2 changes significantly the intensity distribution of the output Airy 1 [Fig. 2(c)]. Moreover, the forward beam 1 intensity shifts faster and further in the −*x*-direction (*x* = −28 *μ*m = −2*x*_*A*_ = *S*) towards the Airy beam 2 main lobe. In the case presented on Fig. 2(c) we inject the CP Airy beam 2 at *t* = 0 s at the position *x* = −2*x*_*A*_ = 28 *μ*m. Due to the nonlinear effect and the large Airy beam transverse dimension, the refractive index variation appears at position *x* = −2*x*_*A*_ < 0 *μ*m, beyond the zero-deflection position of Airy beam 1. By analyzing the single beam case propagation, the time to shift towards the zero-deflection position appears between 200 ms and 400 ms [Fig. 2(b)]; in comparison the intensity has already shifted beyond *x* = −2*x*_*A*_ *μ*m at 200 ms in the CP configuration [Fig. 2(c)]. Due to the higher total intensity inside the crystal when using CP Airy beams, the faster waveguide photoinduction can explain the faster response time.

In what follows, our study will focus on the way to control the photoinduced waveguiding structure using CP Airy beam by varying three parameters: *S* the transverse shift between Airy beams 1 and 2, the beams’ size *x*_*A*_ and the external applied voltage *U*_*SBN*_.

### Influence of the beams’ misalignment: *S*

In previous paragraphs we have shown that adding a CP Airy beam 2 gives the possibility to enhance the shift and the intensity concentration of the single Off Shooting Soliton towards the higher lobe orders. In what follows, we shift the Airy beam 2 main lobe in the opposite direction: *S* = +2*x*_*A*_ = +28 *μ*m and analyze the photoinduced waveguiding structure.

**Figure 3:**
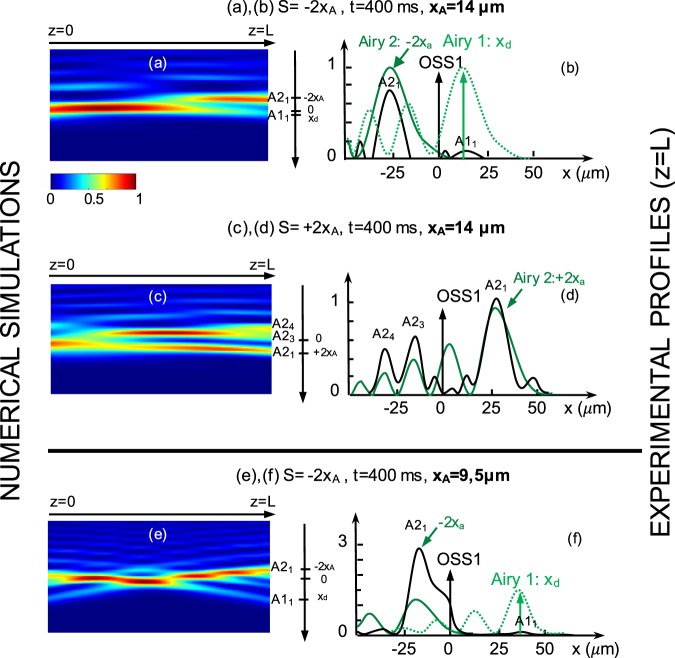
Nonlinear interactions of two CP Airy beams for different misalignments *S* and beam sizes *x*_*A*_ (*P*_*A*_ = 60 *μ*W and *U*_*SBN*_ = 2000 V)]. (**a**,**c**,**e**) Numerical intensity distribution inside the crystal and (**b**,**d**,**f**) experimental transverse intensity profile of the forward output beam at *z* = *L*.

Note that the setup does not allow us to observe and measure inside the nonlinear crystal the optical induced waveguides. We therefore illustrate our experimental measurements with numerical simulations using the model presented in^18^. The numerical simulations presented in Fig. 3(a,c,e) show the total intensity distribution inside the crystal and therefore the corresponding photoinduced waveguiding structure. Similarly to Fig. 2(a), the different outputs reachable by the Airy beam 1 are identified (see the numbering of the outputs): these outputs can be controlled through the input position (transverse shift S) of the Airy beam 2. The first case [Fig. 3(a,b)], where the CP main lobe is beyond the forward zero-deflection position at *x* = −2*x*_*A*_ *μ*m, has been discussed in the previous paragraph. A qualitative comparison between the numerical and experimental output positions (named *A*1_1_, *A*2_1_) shows a good agreement in terms of both the intensity and the position of the outputs. Figure 3(c,d) focus on the second case, where Airy beam 2 is launched in the opposite direction at *x* = +2*x*_*A*_ *μ*m. The numerical insight shows that the self-focusing Airy beam 1 does not propagate through a straight line, but is divided into the CP lobe (*A*2_1_) and the higher lobe orders (*A*2_3_, *A*2_4_). This splitting of the beam suggests beam’s attraction towards both transverse −*x* and +*x*-directions. When we plot the transverse experimental profiles on Fig. 3(d), the experimental measurements confirm the attraction of Airy beam 1 in both directions by the backward Airy beam 2. We observe three output beams possibilities of the transverse intensity profile of the Airy beam 1 at *z* = *L*, respectively separated up to 60 *μ*m. Those output positions do not correspond to the three first lobes of Airy beam 2, but the first, third and fourth lobes. The solitonic zero-deflection output position coinciding with the second backward lobe order does not possess any intensity peak. This striking result is due to the fact that the waveguides at position *A*2_3_, *A*2_4_ have been photoinduced by the less intense third and fourth lobe orders of Airy beam 2. As Airy beam 1 is naturally attracted towards the +*x*-direction, a part of the beam shifts towards the solitonic zero-deflection position (OSS) and pursues its propagation towards the linear higher lobe orders of Airy beam 2. Simultaneously a part of the intensity is shifted to the Airy beam 2 main lobe position at *x* = +2*x*_*A*_, due to the high optical energy contained in this lobe (half of the total optical energy).

In this section, we demonstrated transient shift of a self-focused Airy beam towards the multi-lobe CP Airy beam structure. By adjusting *S*, the separation distance between the CP beams, we observe the attraction either towards the main CP lobe underlying the solitonic structure or towards multiple lobes: this corresponds to a demultiplexing of the soliton into multiple beams. Similarly to numerical simulations presented in ref.^18^, we showed that two counter-propagating Airy beams can induce multiple output waveguiding structures that cannot be achieved with only two counterpropagating Gaussian beams. The resulting waveguiding structure remains possible for higher transverse shifts of CP beams that by far exceed the beam waist (*S* = ±2*x*_*A*_). This unique kind of attraction is enabled by the multi-lobe structure of Airy beams.

### Influence of the Airy beams’ size: *x*_*A*_

It is worth noting that a smaller Airy beam [*x*_*A*_ = 9.5 *μ*m, Fig. 3(f)] is more truncated than a larger one [*x*_*A*_ = 14 *μ*m, Fig. 3(d)] and presents a shorter linear shape-preserving and accelerating propagation distance^5^. We therefore suggest to study the influence of the Airy beam linear deflection on the self-focusing solitonic behavior of the CP Airy beams’ system. As depicted through the parameter *x*_*d*_ on Fig. 3, the smaller Airy beam has more deflected [*x*_*d*_ = 36 *μ*m, Fig. 3(e,f)] than the Airy beam with *x*_*A*_ = 14 *μ*m [Fig. 3(a,b), *x*_*d*_ = 12 *μ*m]. To make the comparison with the previous results, the CP Airy beam is chosen to be beyond the zero-deflection position of the forward beam (*S* = −2*x*_*A*_). When the electric bias voltage *U*_*SBN*_ is switched on at *t* > 0 *s*, we observe an intensity peak towards the CP main lobe [Fig. 3(f)]. By comparison with Fig. 3(a,b), for *x*_*A*_ = 9.5 *μ*m the position of the intensity peak has shifted up to −55 *μ*m [almost six lobe waists compared to 3 lobe waists on Fig. 3(b), −40 *μ*m] and the intensity has increased up to 3 times the linear intensity peak whereas only approximately 75% of the energy is attracted towards the CP main lobe for *x*_*A*_ = 14 *μ*m [Fig. 3(a,b)]. The position of Airy beam 2 in Fig. 3(f), closer to the OSS1 position, makes easier the energy distribution towards the Airy beam 2 main lobe, thus increasing the intensity peak. Depending on the size of the Airy beam, we therefore observe two attractive configurations: (i) small Airy beam (*x*_*A*_ = 9.5 *μ*m), where the CP Airy beam enhances the spatial separation of the Airy-soliton from the linear output [Fig. 3(e,f)], (ii) larger Airy beam (*x*_*A*_ = 14 *μ*m), where multiple CP lobes attract the forward nonlinear propagating Airy beam leading to demultiplexing propagation [1 to 2 outputs, Fig. 3(a)]. As the beam’s size is defined by the SLM-modulation [Fig. 1], the switch between the interaction schemes (i) and (ii) can be computer-controlled in real time.

### Influence of the bias voltage: *U*_*SBN*_

**Figure 4:**
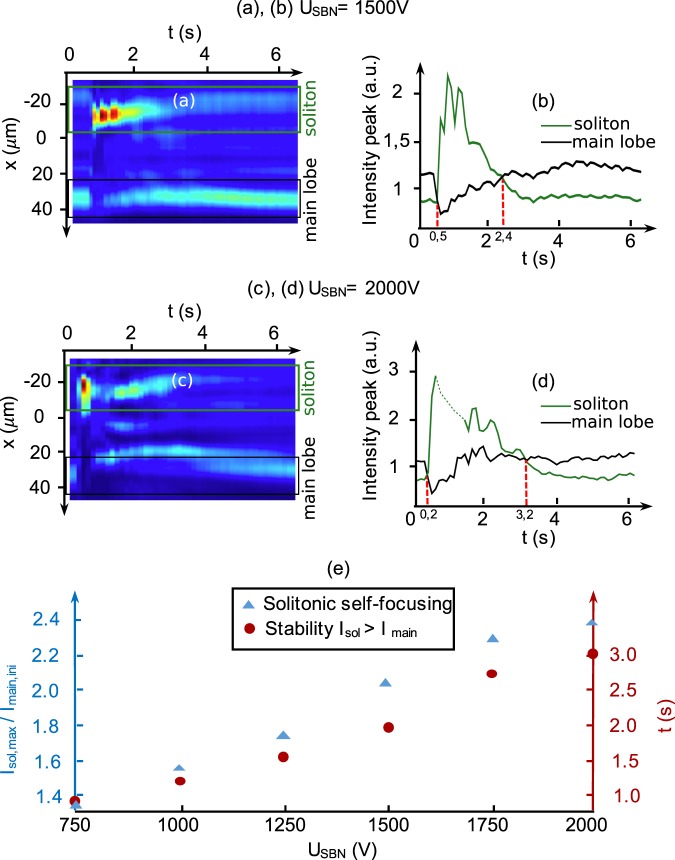
Influence of the external bias voltage for two CP Airy beams with *D* = −2*x*_*A*_, *P*_*A*_ = 60 *μ*W and *x*_*A*_ = 9.5 *μ*m. Along time: (**a**) (resp. (**c**)) experimental transverse intensity distribution of the output beam at *z* = *L* and (**b**) (resp. (**d**)) evolution of the energy around the linear forward main lobe’s position and the solitonic output position (enhanced by the backward main lobe) for *U*_*SBN*_ = 1500 V (resp. *U*_*SBN*_ = 2000 V). (**e**) Evolution of the solitonic peak intensity and its temporal stability for increasing voltages *U*_*SBN*_.

It is worth noting that the solitonic regimes of the CP Airy beams observed in the previous figures are not stationary but relax after few seconds. This spatio-temporal dynamics is illustrated on Fig. 4 in particular for *U*_*SBN*_ = 1500 *V* [Fig. 4(a,b)] and *U*_*SBN*_ = 2000 *V* [Fig. 4(c,d)]. The CP Airy beam 2 is injected at *x* = −2*x*_*A*_ and similarly to Fig. 3(f), the forward output intensity is mainly concentrated around two transverse positions: its linear main lobe’s position *x*_*d*_ and the position of the soliton created from the backward main lobe [green and black frames on Fig. 4(a,c)]. To measure and compare the temporal evolution of both outputs, we integrate the intensity around both transverse locations as depicted on Fig. 4(b,d) (green and black curves). Finally the role of the external electric field is summarized on Fig. 4(e). For increasing values of the nonlinearity (*U*_*SBN*_), this latter enhances two parameters: (i) the solitonic peak energy value [blue plot, Fig. 4(e)] corresponding to the ratio between the solitonic peak intensity (*I*_*sol*,*max*_) and the intensity of the initial Airy main lobe (*I*_*main*,*ini*_) and (ii) the temporal duration of the solitonic output beam [red plot, Fig. 4(e)] corresponding to the period of time when the intensity of the nonlinear soliton (*I*_*sol*_) is beyond the intensity of the linear deflected Airy beam (*I*_*main*_) [see also the red dashed-lines in Fig. 4(b,d)]. The bias voltage presents therefore an interesting tuning parameter to control the strength and the stability of the interaction of the forward self-focusing Airy beam 1 at an output position defined by the CP Airy beam 2. It is worth noting on Fig. 4 that those effects seem to saturate for high nonlinearities (strong *U*_*SBN*_). This is due to the photorefractive effect which is faster (response time) for high nonlinearities [the soliton starts appearing at 200 ms on Fig. 4(d) compared to 500 ms on Fig. 4(b)] but also saturable.

## Conclusion

To conclude we report on the first experiment showing large multi-scale incoherent interactions between two CP Airy beams. By adjusting the nonlinearity strength, the beams’ size, the transverse shift and the external electric field applied, the self-focusing Airy beam is attracted towards the different lobes positions of the CP Airy beam.

The experimental results underline the various and complex waveguiding structures achievable through the interconnection of two CP Airy beams. We presented a number of easy-to-tune parameters that enable to control the attraction and therefore allows the guiding of the forward self-focused Airy beam by controlling the backward CP Airy beam position (single or multiple outputs). The accelerating propagation of the Airy beam enables strong interactions between CP Airy beams even in cases where the transverse shift between the CP Airy main lobes is large: up to six lobe waists in comparison to the two beam waist limit of CP Gaussian beams. In addition to the spatial tuning of the output beam, we can engineer the number of outputs thanks to the multi-lobe structure of the Airy beam (up to three outputs). Finally these solutions can be controlled in time using the bias voltage applied on the nonlinear medium. Most importantly, this large diversity of interactions, and in particular a photoinduced waveguide with multiple discrete outputs is made possible using only two CP Airy beams, i.e. a situation that again contrasts with the performances achieved so far using CP Gaussian beams.

## Data Availability

The datasets generated and analyzed in this paper are available from the corresponding author on reasonable request.

## References

[1] Siviloglou GA, Christodoulides DN (2007). Accelerating finite energy airy beams. Opt. Lett..

[2] Siviloglou, G. A., Broky, J., Dogariu, A. & Christodoulides, D. N. Observation of Accelerating Airy Beams. *Phys. Rev. Lett*. **99**, 213901 http://www.opticsinfobase.org/abstract.cfm?URI=FiO-2007-PDP_B3, 10.1103/PhysRevLett.99.213901 (2007). 10.1103/PhysRevLett.99.213901 18233219

[3] Broky J, Siviloglou GA, Dogariu A, Christodoulides DN (2008). Self-healing properties of optical Airy beams. Opt. Express.

[4] Berry MV, Balazs NL (1979). Nonspreading wave packets. Am. J. Phys..

[5] Morris JE, Mazilu M, Baumgartl J, Cižmár T, Dholakia K (2009). Propagation characteristics of Airy beams: dependence upon spatial coherence and wavelength. Opt. Express.

[6] Jia S, Lee J, Fleischer JW, Siviloglou GA, Christodoulides DN (2010). Diffusion-Trapped Airy Beams in Photorefractive Media. Phys. Rev. Lett..

[7] Kaminer I, Segev M, Christodoulides DN (2011). Self-accelerating self-trapped optical beams. Phys. Rev. Lett..

[8] Lotti A (2011). Stationary nonlinear airy beams. Phys. Rev. A - At. Mol. Opt. Phys..

[9] Hu Y (2012). Reshaping the trajectory and spectrum of nonlinear airy beams. Opt. Lett..

[10] Wiersma N, Marsal N, Sciamanna M, Wolfersberger D (2016). Airy beam self-focusing in a photorefractive medium. Sci. Rep..

[11] Bouchet T, Marsal N, Sciamanna M, Wolfersberger D (2018). Solitonic characteristics of airy beam nonlinear propagation. Phys. Rev. A.

[12] Zhang YY (2013). Soliton pair generation in the interactions of airy and nonlinear accelerating beams. Opt. Lett..

[13] Zhang, Y. Y. *et al*. Interactions of airy beams, nonlinear accelerating beams, and induced solitons in kerr and saturable nonlinear media. *Opt. Express* **22**, 7160–7171, 10.1364/OE.22.007160. arXiv:1403.1952v1 (2014). 10.1364/OE.22.007160 24664064

[14] Shen M, Gao J, Ge L (2015). Solitons shedding from airy beams and bound states of breathing airy solitons in nonlocal nonlinear media. Sci. Rep..

[15] Diebel F, Bokić BM, Timotijević DV, Jović Savić DM, Denz C (2015). Soliton formation by decelerating interacting Airy beams. Opt. Express.

[16] Petrović MS, Belić MR, Denz C, Kivshar YS (2011). Counterpropagating optical beams and solitons. Laser Photon. Rev..

[17] Belić MR, Jander P, Strinić A, Desyatnikov AS, Denz C (2003). Self-trapped bidirectional waveguides in a saturable photorefractive medium. Phys. Rev. E.

[18] Wiersma, N., Marsal, N., Sciamanna, M. & Wolfersberger, D. All-optical interconnects using airy beams. *Opt. Lett*. 39, 5997–6000, OL.39.005997 (2014). 10.1364/OL.39.005997 25361139

[19] Wiersma, N., Photorefractive self-focusing Airy beams: Nonlinear interactions and all-optical waveguiding. *PhD Thesis*, Université de Lorraine (France) (2016).

